# Vimentin Localization in the Zebrafish Oral Cavity: A Potential Role in Taste Buds Regeneration

**DOI:** 10.3390/ijms242115619

**Published:** 2023-10-26

**Authors:** Marialuisa Aragona, Caterina Porcino, Marilena Briglia, Kamel Mhalhel, Francesco Abbate, Maria Levanti, Giuseppe Montalbano, Rosaria Laurà, Eugenia Rita Lauriano, Antonino Germanà, Maria Cristina Guerrera

**Affiliations:** 1 Zebrafish Neuromorphology Lab, Department of Veterinary Sciences, University of Messina, 98168 Messina, Italy; mlaragona@unime.it (M.A.); caterina.porcino@unime.it (C.P.); marilena.briglia@unime.it (M.B.); kamel.mhalhel@unime.it (K.M.); franco.abbate@unime.it (F.A.); mblevanti@unime.it (M.L.); gmontalbano@unime.it (G.M.); laurar@unime.it (R.L.); agermana@unime.it (A.G.); 2 Department of Chemical, Biological, Pharmaceutical and Environmental Sciences, University of Messina, 98166 Messina, Italy; eugenia.lauriano@unime.it

**Keywords:** zebrafish, oral cavity, taste buds, Vimentin RV202, Calretinin N-18, SEM

## Abstract

The morphology of the oral cavity of fish is related to their feeding habits. In this context, taste buds are studied for their ability to catch chemical stimuli and their cell renewal capacity. Vimentin RV202 is a protein employed as a marker for mesenchymal cells that can differentiate along different lineages and to self-renew, while Calretinin N-18 is employed as a marker of sensory cells, and ubiquitin is a protein crucial for guiding the fate of stem cells throughout development. In this study, a surface morphology investigation and an immunohistochemical analysis have been conducted. The results of the present study reveal, for the first time, the presence of Vimentin RV202 in a taste bud cell population of zebrafish. Some taste bud cells are just Vimentin RV202-immunoreactive, while in other cells Vimentin RV202 and Calretinin N-18 colocalize. Some taste buds are just reactive to Calretinin N-18. Vimentin RV202-immunoreactive cells have been observed in the connective layer and in the basal portion of the taste buds. The immunoreactivity of ubiquitin was restricted to sensory cells. Further studies are needed to elucidate the role of Vimentin RV202 in the maturation of taste bud cells, its potential involvement in the regeneration of these chemosensory organs, and its eventual synergic work with ubiquitin.

## 1. Introduction

The sense of taste enables vertebrates to detect nutritious and/or potentially harmful or toxic substances. The study of the morphology of the oral cavity of fish gives indications about their diet, capacity, and the modality of feeding. It reflects the morpho-functional adaptations of fish to the environment and plays many roles related to food intake and chemical detection of environmental changes [1]. The detection of chemical stimuli is carried out by taste buds. Therefore, the presence, morphology, abundance, and distribution of taste buds, have been studied in several species of fish [2,3,4]. This kind of study shows a strict comparative correlation with other vertebrates. Taste buds are made up of taste receptor cells that detect and convey taste information. The identification of these chemosensory cells is possible thanks to specific markers, such as Calretinin N-18 and S100 [5]. Like other sensory cells in fish, taste bud cells are continually and rapidly renewed [6], but little is known about the nature of their renewal [7]. To better understand this phenomenon, neurotrophins and their specific receptor localization have been investigated given their role in sensory organs neuron survival, differentiation proliferation, and development [8,9,10]. Therefore, to keep studying the oral cavity of zebrafish, and its taste buds, Vimentin RV202 localization in these chemosensory organs has been investigated in the present study. Vimentin RV202 is instrumental in physiological key processes, such as normal cellular homeostasis, inflammation, apoptosis, tissue integrity, and nerve injury [11,12,13], and is the major cytoskeletal component of mesenchymal cells [14]. However, Vimentin RV202 has been also found in “borderline” epithelia that interface with underlying connective tissue, so some authors assume the involvement of this protein in particular events in development, cell migration, or even dedifferentiation [15]. Vimentin RV202 is conserved during evolution, and its DNA and aminoacidic sequences are very similar between fish and mammals [16,17,18,19]. This protein has been found in some fibroblast-like cells of the tongue of two adult fish species, *Salmo salar* and *Oncorhynchus mykiss,* respectively [3]. Moreover, Vimentin RV202 has been found in the taste buds of other vertebrates [20]. It has also been shown that Vimentin RV202 interacts with several signaling proteins, including ubiquitin [21]. Ubiquitin is a structurally conserved protein that in eukaryotic cells regulates several processes. Ubiquitin could be found either covalently joined to a wide range of cytoplasmic and nuclear proteins or free. Recent genetic and biochemical evidence demonstrates that ubiquitin conjugation of short-lived proteins is crucial for their selective in vivo degradation. The supposed roles of the ubiquitin system in the stress response and chromosome function have also been discussed [22]. Ubiquitin and its relatives covalently attach to other cellular proteins to carry out their functions, therefore changing the stability, localization, and/or activity of the target protein [23]. Modification with ubiquitin is crucial for many essential signaling networks guiding the fate of stem cells throughout development. Ubiquitin ligases are often expressed in specific kinds of cells and/or at particular developmental stages, and mutations in genes encoding these proteins are related to diseases caused by anomalous stem cell functioning [24]. Although Reutter and Witt [25] hypothesized the presence of Vimentin RV202 in the taste buds of fish, according to our knowledge, Vimentin RV202 localization in zebrafish taste buds has never been investigated before. Thanks to its lower costs and less time-consuming experiments, the zebrafish model has become an attractive alternative to rodents. Zebrafish is a consolidated experimental model in embryology, cell biology, and translational medicine studies, thanks to the possibility of creating many mutant phenotypes resembling human clinical disorders [26,27]. Indeed, it has been used in experiments relating to taste and odor detection [28,29], as well as hearing and deafness [30], and for assessing new drugs [31]. As a model, zebrafish presents many methodological advantages, and collections of resources are available. This model allows the implementation of methodologies for modulating gene expression, behavioral tests to examine changes in motor activity, and the employment of tools applicable in vivo, such as whole-mount imaging, thanks to the optical transparency of zebrafish embryos and early larval stages, and it makes simultaneous chemical/drug testing on a large number of animals simple [32]. Finally, the fully sequenced genome is available [33]. Therefore, the present study aimed to investigate Vimentin RV202 localization in the taste buds of adult zebrafish together with Calretinin N-18 which is employed to mark sensory cells and ubiquitin.

## 2. Results

### 2.1. Ultrastructure and Histology of Taste Buds in the Zebrafish Model

**Figure 1:**
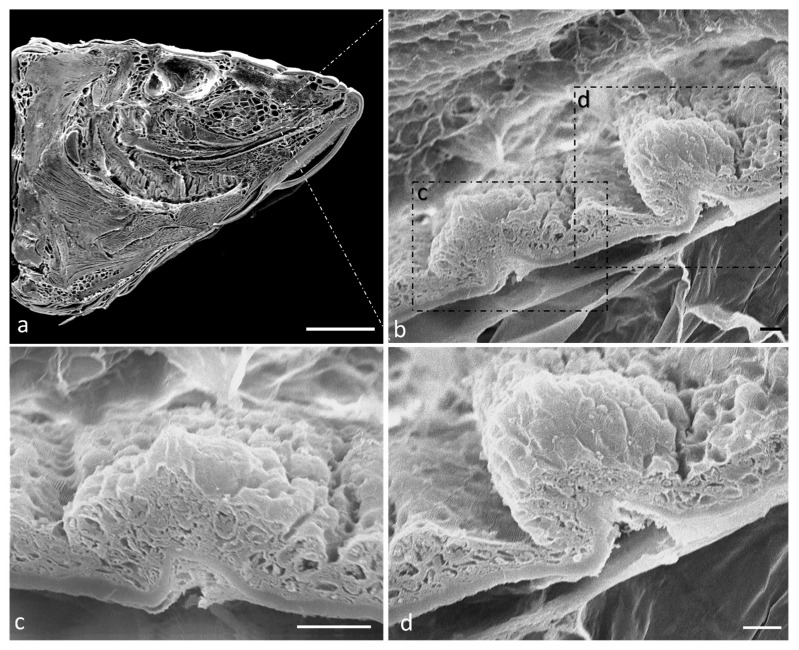
(**a**) Scanning electron microscope micrograph of a sagittal section of a zebrafish head. (**b**) Scanning electron microscope of two taste buds with different shapes. Higher magnification of onion-shaped taste bud (**c**) and pear-shaped taste bud (**d**). Scale bar: 1 mm (**a**) 10 μm (**b**) 20 μm (**c**) 10 μm (**d**).

Scanning electron microscope analysis of a sagittal section of a zebrafish head shows that the oral cavity has a folded mucosa with scatted onion- and pear-shaped taste buds (Figure 1a–d).

**Figure 2:**
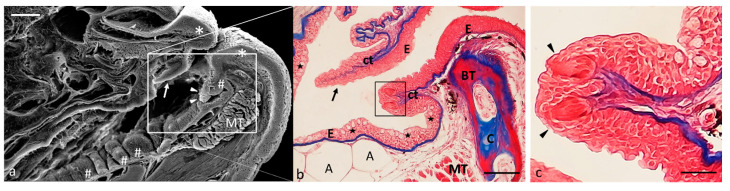
(**a**) Scanning electron microscope micrograph of a median section of a zebrafish head. Well-developed lips (asterisks), semilunar valve (arrow), taste buds (arrowheads), mucosal folds (#), muscular tissue (MT). (**b**) Light micrograph (Masson’s trichrome with aniline blue staining): taste buds (inset), semilunar valve (black arrow). Stratified epithelium (E); connective tissue (ct); mucous cells (stars); adipocytes (A); bone (BT); cartilage (C); muscular tissue (MT). (**c**) High magnification of taste buds (arrowheads) of inset in image. Scale bar: 1 mm (**a**). Magnification 10×, scale bar 100 µm (**b**). Magnification 63× oil, scale bar 10 µm (**c**).

The oral cavity is limited by two well-developed lips. Each lip has two semilunar valves, but the valve of the lower lips is more developed and delimits the oral cavity rostrally. The floor of the mouth shows several folds sprinkled with taste buds. Taste buds are also present in the oral vault and on the dorsal surface of the tongue (Figure 2a,b).

**Figure 3:**
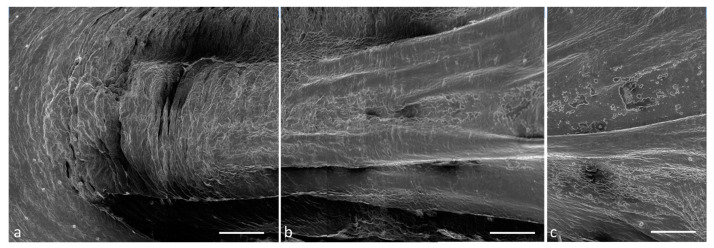
Scanning electron microscope micrograph of the dorsal surface of the tongue: (**a**) apex, (**b**) body, (**c**) root. Scale bar: 1 mm (**a**–**c**).

The tongue has a triangular pyramidal shape and presents an apex (Figure 3a), a body (Figure 3b), and a root (Figure 3c).

**Figure 4:**
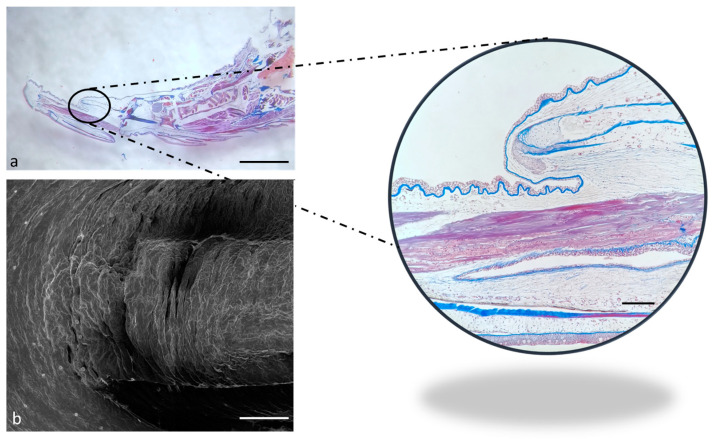
(**a**) Stereomicrograph of a sagittal section of the tongue (Masson trichrome with aniline blue staining): apex of the tongue (circular insert). (**b**) Scanning electron microscope micrograph of the tongue apex. Magnification 4×, scale bar 1 mm (**a**). Scale bar: 1 mm (**b**). Magnification 10×, scale bar 200 µm (insert).

The apex appears rostrally merged to the floor of the mouth under the SEM (Figure 4b), while it seems partially free under the optical microscopy (Figure 4a, insert).

**Figure 5:**
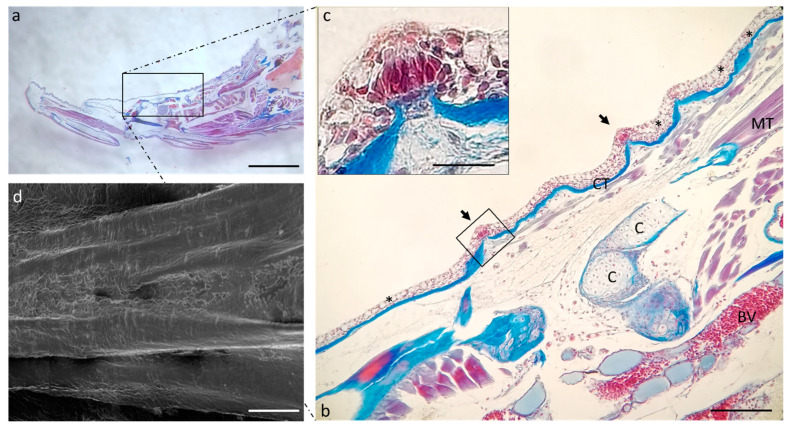
(**a**) Stereomicrograph of a sagittal section of the tongue: dorsal surface of the tongue body (insert). (**b**) Light micrographs of the dorsal surface of the tongue with taste buds (arrows). Connective tissue (CT); cartilage (C); muscular tissue (MT); blood vessel (BV); goblet cells (asterisks). (**c**) high magnification of taste bud in image. (**b**) Masson trichrome with aniline blue staining. (**d**) Scanning electron microscope micrograph of the tongue body. (**a**) Magnification 4×, scale bar 1 mm. (**b**) Magnification 10×, scale bar 200 µm. (**c**) Magnification 63× oil, scale bar 10 µm. (**d**) Scale bar: 1 mm.

**Figure 6:**
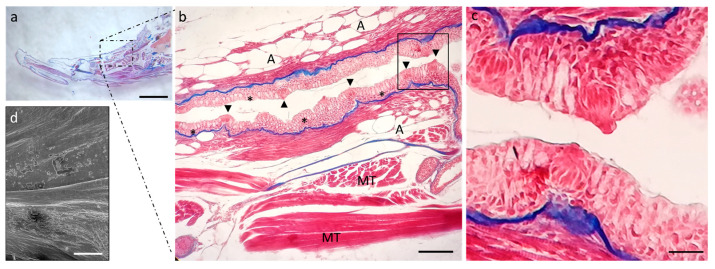
(**a**) Stereomicrograph of a sagittal section of the tongue: dorsal surface of the tongue root (insert). (**b**) Light micrographs of the dorsal surface of the tongue root showing the lingual mucosa with several goblet cells (asterisks) and taste buds (arrowheads). Muscular tissue (MT), adipocites (A); Masson trichrome with aniline blue staining. (**c**) high magnification of taste buds in (**b**). (**d**) Scanning electron microscope micrograph of the tongue body. (**a**) Magnification 4×, scale bar 1 mm. (**b**) Magnification 10×, scale bar 200 µm. (**c**) Magnification 63× oil, scale bar 10 µm. (**d**) Scale bar: 1 mm.

Proceeding in the aboral direction, it is possible to observe the scattered tastebuds on the entire dorsal surface of the tongue body (Figure 5b) and root (Figure 6b). In the floor of the mouth, goblet cells are numerous and intercalated in the lining epithelium of the lingual mucosa (Figure 6b).

### 2.2. Calretinin (N-18) and Vimentin RV202 Specificity in Zebrafish

The alignment of the Calretinin N-18 sequences of the human CAA39991.1 and those of rats, mice, and zebrafish shows that the proteins have 98.52, 98.15 and 71.96% of amino acids match exactly (identity), respectively (Table 1), defined either by their chemical properties or based on a point accepted mutation matrix, between the different sequences. The comparable identity of zebrafish Calretinin N-18 to the rat and mice protein sequences allows us to hypothesize that the used commercial antibody could work on zebrafish.

### 2.3. Vimentin RV202 and Calretinin N-18 Colocalize in Some Taste Buds of Zebrafish

**Figure 7:**
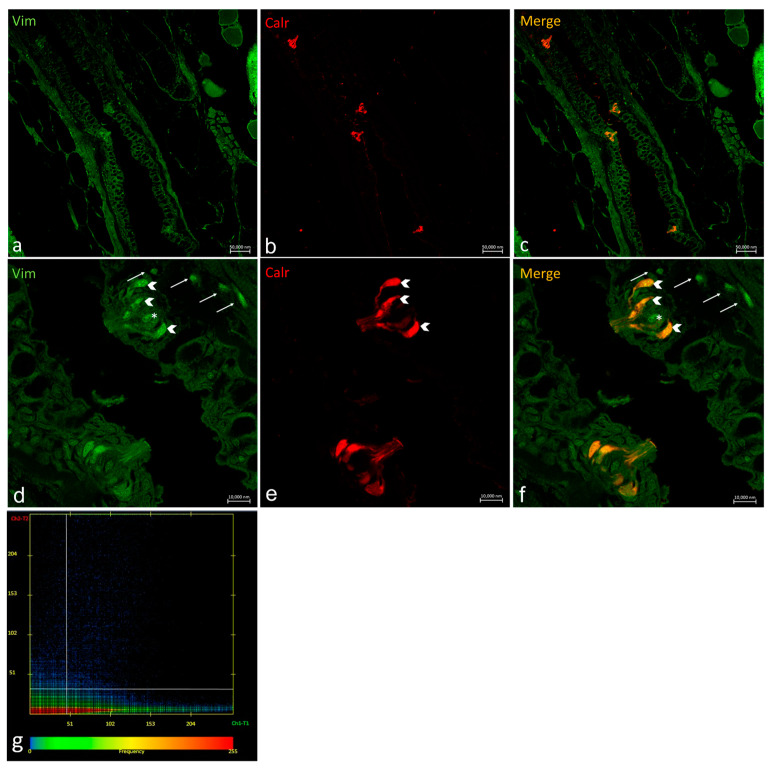
Oropharyngeal taste buds. Immunohistochemical detection (immunofluorescence method) of Vimentin RV202 (**a**,**d**) and Calretinin N-18 (**b**,**e**). Immunohistochemical detection of Vimentin RV202 and Calretinin N-18 in colocalization view (**c**,**f**). Vimentin RV202-immunoreactive cells in the connective layer (arrows). Vimentin RV202-immunoreactive cells interspersed between taste bud sensory cells (asterisks). Taste bud sensory cells (gallon arrows). (**a**–**c**) Magnification 10×; scale bar 50 µm. (**d**–**f**) Magnification 63×; scale bar 10 µm. (**g**) Scatter plot of Vimentin RV202/ Calretinin N-18 colocalization in zebrafish taste buds. The X-axis and Y-axis indicate the immunofluorescent signal, respectively, for Calretinin N-18 and Vimentin RV202. The intersection point between two lines indicates colocalization, which means the localization of Calretinin N-18 and Vimentin RV202 in the same cell. Zen 2011 (LSM 700 Zeiss software ZEN 3.7).

Oropharyngeal taste buds show different patterns of distribution for Vimentin RV202 and Calretinin N-18 (Figure 7). Some taste bud cells are just Vimentin RV202-immunoreactive, while in other cells Vimentin RV202 and Calretinin N-18 colocalize. Some taste buds are just reactive to Calretinin N-18 (Figure 7c,f). Vimentin RV202-immunoreactive cells have been also observed in the underlying connective layer and in the basal portion of the taste buds (Figure 7d,f).

**Figure 8:**
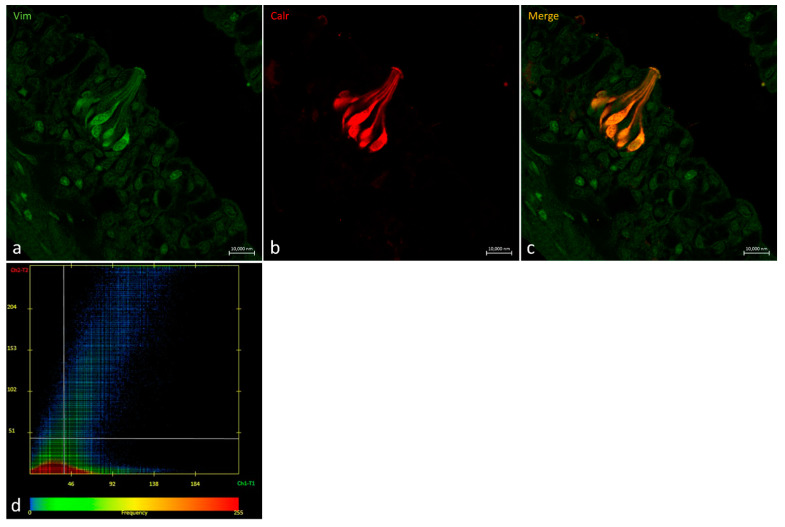
Oropharyngeal taste bud. (**a**) Immunohistochemical detection (immunofluorescence method) of Vimentin RV202 and (**b**) Calretinin N-18. (**c**) Immunohistochemical detection of Vimentin RV202 and Calretinin N-18 in colocalization view. (**a**–**c**) Magnification 63×; scale bar 10 µm. (**d**) Scatter plot of Vimentin RV202/ Calretinin N-18 colocalization in zebrafish taste buds. The X-axis and Y-axis indicate the immunofluorescent signal, respectively, for Calretinin N-18 and Vimentin RV202. The intersection point between two lines indicates colocalization, which means the localization of Calretinin N-18 and Vimentin RV202 in the same cell. Zen 2011 (LSM 700 Zeiss software ZEN 3.7).

Vimentin RV202 and Calretinin N-18 colocalized in cells having the longitudinal axis positioned toward the free tongue surface (Figure 8).

**Figure 9:**
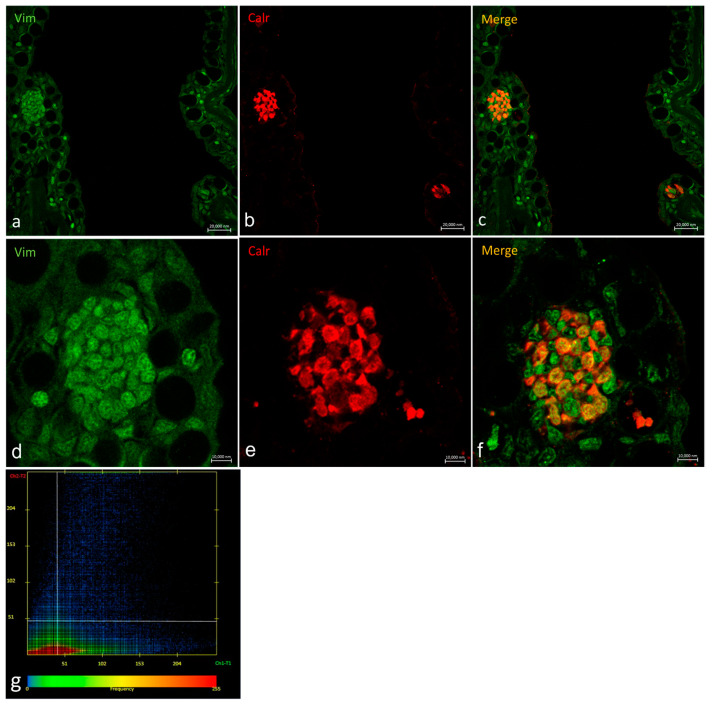
Transversal section of the basal part of a taste bud. (**a**,**d**) Immunohistochemical detection (immunofluorescence method) of Vimentin RV202 and (**b**,**e**) Calretinin N-18. (**c**,**f**) Immunohistochemical detection of Vimentin RV202 and Calretinin N-18 in colocalization view. (**a**–**c**) Magnification10×; scale bar 20 µm. (**d**–**f**). Magnification 63×; scale bar 10 µm. (**g**) Scatter plot of Vimentin RV202/ Calretinin N-18 colocalization in zebrafish taste buds. The X-axis and Y-axis indicate the immunofluorescent signal, respectively, for Calretinin N-18 and Vimentin RV202. The intersection point between two lines indicates colocalization, which means the localization of Calretinin N-18 and Vimentin RV202 in the same cell. Zen 2011 (LSM 700 Zeiss software ZEN 3.7).

In a transversal section of the basal part of a taste bud, it is possible to observe Vimentin RV202-immunoreactive cells between the Calretinin N-18-immunoreactive cells and scattered cells where the two antibodies colocalize (Figure 9).

### 2.4. Ubiquitin-Immunoreaction in Taste Buds

**Figure 10:**
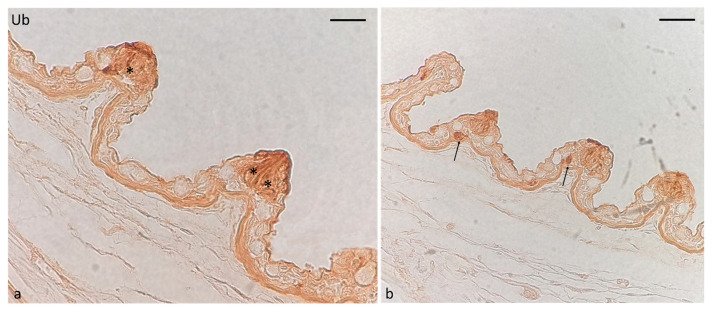
Immunohistochemical localization of ubiquitin immunoreactivity in oral taste buds of adult zebrafish. (**a**) Sensory cells (asterisks). (**b**) Isolated cells (arrows) near taste buds. Magnification: 60× (**a**); 40× (**b**).

The immunoreactivity for ubiquitin in oropharyngeal taste buds was restricted to cells that are recognized as sensory cells for their shape and position (Figure 10a). However, immunoreactivity was also detected in isolated cells near taste buds (Figure 10b).

**Figure 11:**
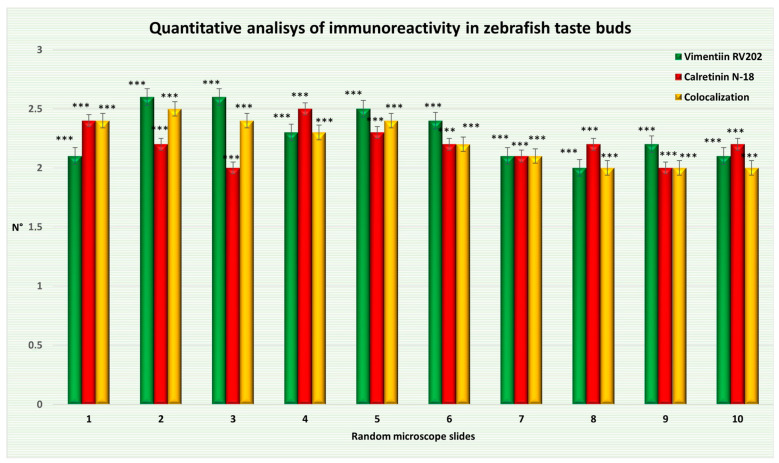
Graphical representation of adult zebrafish taste bud cells immunoreactive to Vimentin RV202, Calretinin N-18, and cells with two proteins colocalized. The statistical analysis shows a distribution pattern of the two proteins. Data represent the average of measurements from random fields of ten sections. N°: mean of taste bud cells immunopositive to Vimentin RV202, Calretinin N-18, and colocalized. Statistical significance: *** *p* < 0.001.

What emerges from our observations is that some taste bud cells are Vimentin RV202 and Calretinin N-18 immunoreactive. Just a subpopulation of cells in some taste buds showed colocalization of Vimentin RV202 and Calretinin N-18, which is a marker of sensory cells. As a matter of fact, the statistical analysis shows a distribution pattern of the two proteins in adult zebrafish taste buds. The average of taste bud cells only Vimentin RV202-positive, the average of cells only Calretinin N-18-positive, and the average of cells showing only colocalization are shown in Figure 11 and Table 2.

## 3. Discussion

This study aimed to contribute to the scientific literature regarding the zebrafish oral cavity and reports how Vimentin RV202 and Calretinin N-18 colocalize in the taste buds of its oral cavity. In addition, ubiquitin localization in these sensory organs has been investigated too. In general, the study of the oral cavity is relevant to understanding the correlations between its morphology and fish feeding habits. Oral cavity morphology could play many roles in the detection of environmental changes [1]. In particular, this kind of detection happens because taste buds are involved in different fish behaviors, such as feeding, reproduction, and migration [34]. Taste bud cells are epithelial cells and not nerve cells, despite their sensory nature. These specialized epithelial cells detect chemical information and transmit it directly to the central nervous system (CNS) by bipolar neurons. Taste buds consist of modified cells of different types, described as dark cells and light cells according to their electron density in the transmission electron microscope (TEM) [35,36]. Between these cells, there are basal cells, which resemble Merkel cells but without, as far as known, the staminal or regenerative properties that have been found in the basal cells of mammals [25]. Finally, marginal cells envelop the taste buds and constitute the interface between the sensory epithelium of the organ and other cells of the squamous stratified epithelium. Interest in fish taste buds is raised among researchers because of their capacity to regenerate both through a physiological process of turnover and after damage [37]. The suspension of the regeneration process can cause an altered sense of taste [37]. In mammals, the basement membrane, which is located near the lingual stem cells outside of the taste buds, generates taste bud cells by being actively involved in the cell cycle [37]. In mice, strong evidence demonstrates the origin of taste bud progenitors from local epithelium. However, it is not clear if taste buds progenitors just originate from the adjacent epithelium [38,39,40,41]. According to some authors, in humans, the surrounding epithelial cells may contribute to the development of taste buds, and Vimentin RV202 immunoreactivity is expressed mainly in border (marginal) epithelial cells of taste bud primordia [20]. However, some evidence highlights that, among different taxa, the process leading to taste buds differentiation from source tissue may vary. For instance, in chicks, mesenchymal cells may be contributing sources of developing taste buds [20]. What we found in the present study is that some taste bud cells are Vimentin RV202-immunoreactive. Just a subpopulation of cells in some taste buds showed colocalization of Vimentin RV202 and Calretinin N-18, which is a marker of sensory cells [5]. Vimentin RV202 is a cytoskeletal protein belonging to the intermediate filament family (reviewed by Fuchs and Weber) [42], and it was long considered a specific marker of mesenchymal cells, forming a distinctive type of intermediate filament (IF) in fibroblasts and other related cells [43,44]. Vimentin RV202 was identified in epithelial cells and neurons during morphogenetic movements in embryos and cell migration [45,46,47]. Vimentin RV202-reactive cells have been found also in the tissue underlying the taste buds. This result is consistent with other reports [20,48], showing that the connective tissue underlying taste buds has Vimentin RV202-labeled cells. Indeed, the presence of cells positive for Vimentin RV202 was already detected in other teleost tongues [3]. The presence of Vimentin RV202-reactive cells with the same shape, both in the taste buds and in the underlying tissue, could be explained by the migration of these cells from the connective tissue to the organs to generate new taste bud cells. Indeed, it has been already demonstrated that Vimentin RV202 regulates the formation of cell extensions and controls cell migration through connective tissues [49]. It has been established that the connection between plasma membrane and cytoskeleton, and in particular Vimentin RV202 filaments, is likely to be significant in many adhesion-dependent cell functions [50]. Even if, according to some authors, in teleostean fishes replacement of taste bud cells has been shown to occur from adjacent epithelial cells [51], the trans-differentiation from mesenchymal to epithelial properties, and vice versa, is a well-known phenomenon [20,52,53]. This biological event is a process characterized by cellular morphology, polarity, and motility changes [53], and the gaining of mesenchymal marker expression [54]. Epithelial-to-mesenchymal and mesenchymal-to-epithelial transitions occur both during the pathogenesis of diseases and in normal tissue and organ development. Mesenchymal–epithelial transition (MET) occurs during epithelial damage/repair mechanism and remodeling events. MET has already been demonstrated to be involved in epithelial regeneration during the physiological cellular turnover in different anatomical districts [55,56]. Moreover, MET is the phenomenon responsible for the activation of the terminal differentiation of the progenitor cells during local tissue demand [57]. According to some authors, MET could explain Vimentin RV202 expression in taste bud cells during proliferation and replacement [20,52]. On the other hand, the occurrence of ubiquitin in taste bud cells could suggest the involvement of this protein in MET process regulation in synergy with Vimentin RV202. In general, it is known that progenitor cells show high levels of proteasome activity led by ubiquitin. Such activity is required for progenitor cells self-renewal, pluripotency, and differentiation [58]. Moreover, studies on differentiation programs of mesenchymal stem cells, including neurogenesis, propose that ubiquitin, through its ligases, is involved in cell differentiation [59]. It has been demonstrated that the segregation of ubiquitin during cell division in the course of development processes can change. For instance, in *D. melanogaster* embryos, in mammalian stem cell populations in vitro, and in young mouse neuronal stem cells, segregation of ubiquitin in late stages of mitosis is asymmetric and this asymmetry is reduced in older cells. The same asymmetry has been found for Vimentin RV202 during immunohistochemistry investigation and it has been demonstrated not only that Vimentin RV202 has an asymmetric distribution during late mitosis, but also that Vimentin RV202 co-segregated with ubiquitin the majority of the time. Thus, some authors asked whether asymmetric segregation of Vimentin RV202 and ubiquitin in young dividing cells is associated with cell cycle length [60]. Calretinin N-18-immunoreactivity was detected too. Calretinin N-18 is a member of the hexa-EF-hand protein family. It is mainly expressed in specific neurons of the central and peripheral nervous system, although its expression also occurs in non-neuronal cells [61]. The evidence that some taste buds are immunoreactive for Calretinin N-18 but not to Vimentin RV202 could be a consequence of a different pattern of protein localization in different stages of taste buds’ development or regeneration. This is in accordance with Venkatesan’s et al. [48] results suggesting that a distinct population of taste bud cells that are labeled by different molecular markers might represent different cell types or different phases of taste bud cell development. It has been demonstrated that certain progenitrices cells progressively decrease their Vimentin RV202 content during the differentiation process because it is later replaced by tissue-specific intermediate filaments in most cell types [62]. It remains to be clarified how this replacement takes place and if takes place in fish taste buds. According to the current knowledge, ubiquitinated or damaged proteins reduce the rate of proliferation [60]. Hence, since already differentiated cells have a reduced proliferation rate, it remains unclear if the occurrence of ubiquitinated protein could represent a parameter to evaluate distinct differentiation cell stages. To sum up, it could be hypothesized that different patterns of distribution of Vimentin RV202 and Calretinin N-18 could occur in different cell differentiation phases during the Mesenchymal–epithelial transition, which is characterized by the loss of the mesenchymal marker and the gain of the epithelial marker.

The present study shows, for the first time, the occurrence of Vimentin RV202 in a cell population of taste buds of zebrafish. According to the results of the present study, cells in the connective tissue underlying taste buds are Vimentin RV202-immunoreactive too. These cells could migrate from the connective tissue to the taste buds to take part in their physiological turnover. Vimentin RV202 could play a role in this migration process by mediating cytoskeletal organization. Moreover, immunoreactivity to ubiquitin in taste bud cells and neighboring cells could suggest this protein plays a role in cellular differentiation and specialization. The present results could open new prospects in the field of cellular regeneration investigation in teleosts and the field of translational studies. However, further studies are needed to elucidate the role of Vimentin RV202 in taste bud sensory cells, its potential involvement in the regeneration process triggered by a damage repair mechanism, and its potential interaction with ubiquitin.

## 4. Materials and Methods

The specimens of adult zebrafish (*D. rerio*) have been maintained using routine procedures [8,9,63]. All animal handling protocols were carried out in accordance with the principles outlined in the declaration of Helsinki and approved by the Italian Ministry of Health (A.M. n. 505/2023-PR).

### 4.1. Optical Microscopy

Tissue samples of fresh specimens were fixed in 4% paraformaldehyde in phosphate-buffered saline (PBS) 0.1 M (pH 7.4) for 12–18 h, dehydrated, and routinely embedded in paraffin. For this study, sagittal serial sections 7 μm thick were collected using a Leica RM 2135 microtome (Leica microsystems Nussloch GmbH), mounted on microscope slides, and processed for Masson trichrome with aniline blue staining. A Leica DMRB light microscope with a Leica MC 120 HD camera (Leica Application Suite LAS V4.7) was used to analyze the stained sections (see Table 3 for information on the chemicals used in this study).

### 4.2. Scanning Electron Microscopy

Zebrafish samples were fixed in 0.1 M of Sorensen phosphate buffer with 2.5% glutaraldehyde. The samples were dehydrated in a graded alcohol series (from 50° to 100°, 1 h for each step) after many rinses in the same phosphate buffer, critical point dried in a Balzers CPD 030, and then sputter-coated with 3 nm gold in a Balzers BAL-TEC SCD 050. A scanning electron microscope equipped with a Zeiss EVO LS 10 (Carl Zeiss NTS, Oberkochen, Germany) and operating at a 20 kV accelerating voltage was used to evaluate the processed samples (see Table 3 for information on the chemicals used in this study).

### 4.3. Calretinin (N-18) and Vimentin RV202 Specificity in Zebrafish

Calretinin (N-18) (sc-11644 Santa Cruz Biotechnology) has been declared to be raised against a peptide mapping at the N-terminus of Calretinin N-18 of human origin. Moreover, the datasheet reports that the antibody is predicted to work with mouse, rat, and human samples. In order to understand if the used primary antibody, raised against the human protein, could also work in zebrafish, a protein alignment has been performed for the human, mouse, rat, and zebrafish. The online software NCBI blastp (protein–protein BLAST) was used for peptide alignment [64]. According to the manufacturer, the Vimentin monoclonal antibody (RV202) (cat #OMA1-06001 Thermo Fisher Scientific) used in this study is specifically reactive in zebrafish.

### 4.4. Confocal Immunofluorescence

Sagittal serial sections 10 μm thick were deparaffinized and rehydrated, and finally washed in phosphate-buffered saline (PBS). The sections were first permeated with 0.1% Triton X100 in a PBS solution before being exposed to 0.3% hydrogen peroxide (H_2_O_2_), to prevent the activity of endogenous peroxidase. The slides were rinsed several times in PBS and blocked in 25% fetal bovine serum albumin for 1 h. The sections were treated overnight at 4 °C in a humid chamber with antibodies Vimentin monoclonal antibody (RV202) was used in double-label experiments with a polyclonal antibody to Calretinin-N18 goat antibody (details of the primary antibody are shown in Table 4). After rinsing in PBS solution, the sections were incubated for 1 h at 4 °C with Alexa Fluor 488 donkey anti-Mouse IgG (H+L) and Alexa Fluor 594 donkey anti-Goat IgG (H+L) at room temperature in a dark humid chamber (the details of the secondary antibody are shown in Table 4). To prevent photobleaching, the sections were washed, and mounted with Fluoromount Aqueous Mounting Medium, and then the cover slipped. Sections were analyzed and images were acquired using a Zeiss LSMDUO confocal laser scanning microscope LSM700 AxioObserver (Carl Zeiss Micro Imaging GmbH, Germany). Zen 2011 (LSM 700 Zeiss software ZEN 3.7) built in “colocalization view” [65]. Rapid acquisition of each image was used to reduce photodegradation. Control experiments excluding incubation with primary antibodies were performed (see results in Supplementary Materials) (see Table 3 for information on the chemicals used in this study).

### 4.5. Immunoperoxidase Method

Some serial sections 10 μm thick were deparaffinated and rehydrated, and rinsed in Tris-HCl buffer (0.05 M, pH 7.5) containing 0.1% bovine serum albumin and 0.2% Triton X100. Endogenous peroxidase activity and non-specific binding were blocked (0.3% H_2_O_2_ and 50% fetal bovine serum, respectively), and sections were incubated overnight with a primary antibody specific for ubiquitin. Then, sections were rinsed in the same buffer and incubated for 1.5 h at room temperature with goat anti-rabbit immunoglobulins (IgG) peroxidase-conjugated secondary antibody (details of the primary and secondary antibodies are shown in Table 4). The immunolabeling was displayed using 3–3′ diaminobenzidine (DAB) as a chromogen. Control experiments excluding incubation with primary antibodies were carried out (see results in Supplementary Materials). The slides were analyzed under a Leicia DMRB light microscope equipped with a Leica MC 120 HD camera (Leica Application Suite LAS V4.7) (see Table 3 for information on the chemicals used in this study). 

### 4.6. Statistical Analysis

ImageJ software (Version 1.53t) was used to measure the microscope area collected at random. One-way ANOVA was employed to investigate the statistical significance of the number of taste bud cells detected by Vimentin RV202, Calretinin N-18, and colocalized. SigmaPlot version 14.0 (Systat Software, San Jose, CA, USA) was used for the statistical analysis. An unpaired Z test was also performed. The data was provided as mean values with standard deviations (Δσ). Values of *p* below 0.05 were judged statistically significant in the following order: *** *p* < 0.001, ** *p* < 0.01, and * *p* < 0.05.

## 5. Conclusions

For the first time, the current study documents the presence of Vimentin RV202 in a cell population of taste buds of zebrafish. According to the results of the present study, cells in the connective tissue underlying taste buds are Vimentin RV202-immunoreactive too. These cells could migrate from the connective tissue to the taste buds to take part in their physiological turnover. Vimentin RV202 could play a role in this migration process by mediating cytoskeletal organization. Moreover, immunoreactivity to ubiquitin in taste bud cells and neighboring cells could suggest that this protein plays a role in cellular differentiation and specialization. The abovementioned results could reshape the way the regeneration process is studied in both teleosts and in the field of translational medicine. However, further studies are needed to elucidate the role of Vimentin RV202 in taste bud sensory cells, its potential involvement in the regeneration process triggered by a damage repair mechanism, and its interaction with ubiquitin.

## Figures and Tables

**Table 1:** Comparison of Calretinin N-18 identity between human, mouse, rat, and zebrafish.

Taxa	Max Score	Total Score	Query Cover	E Value	Per. Ident	Acc. Len	Accession
Calretinin N-18							
*Homo sapiens*	-	-	-	-	-	271	CAA39991.1
*Rattus norvegicus*	547	547	100%	0.0	98.52%	271	Query_41808
*Mus musculus*	546	546	100%	0.0	98.15%	271	Query_41807
*Danio rerio*	400	400	100%	7 × 10^−147^	71.96%	269	AAH59467.1

**Table 2:** Mean data ± standard deviation (Δσ) of taste bud cells v immunoreactive, Calretinin N-18 immunoreactive, and cells showing colocalization. The statistical analysis shows a distribution pattern of the two proteins. All features were evaluated per 174.286 ± 3.082 μm (mean). Statistical significance: *** *p* < 0.001.

Random Microscope Slide	Mean ± Δσ of Taste Bud Cells Immunopositive to Vimentin RV202	Mean ± Δσ of Taste Bud Cells Immunopositive to Calretinin N-18	Mean ± Δσ of Taste Bud Cells Showing Colocalization
(1)	2.1 ± 0.7 ***	2.4 ± 0.66 ***	2.4 ± 0.8 ***
(2)	2.6 ± 0.48 ***	2.2 ± 0.6 ***	2.5 ± 0.67 ***
(3)	2.6 ± 0.48 ***	2 ± 0.63 ***	2.4 ± 0.66 ***
(4)	2.3 ± 0.64 ***	2.5 ± 0.67 ***	2.3 ± 0.64 ***
(5)	2.5 ± 0.67 ***	2.3 ± 0.64 ***	2.4 ± 0.8 ***
(6)	2.4 ± 0.8 ***	2.2 ± 0.87 ***	2.2 ± 0.74 ***
(7)	2.1 ± 0.7 ***	2.1 ± 0.83 ***	2.1 ± 0.7 ***
(8)	2 ± 0.63 ***	2.2 ± 0.74 ***	2 ± 0.63 ***
(9)	2.2 ± 0.4 ***	2 ± 0.77 ***	2 ± 0.63 ***
(10)	2.1 ± 0.7 ***	2.2 ± 0.74 ***	2 ± 0.77 ***

**Table 3:** Chemicals used in this study.

Chemicals	Supplier	Dilution	Catalog Number
Paraformaldehyde	Sigma-Aldrich, Inc., St. Louis, MO, USA	4%	158127
Phosphate-buffered saline (PBS)	Sigma-Aldrich, Inc., St. Louis, MO, USA.	manufacturer’s notice	P4417
Triton X100	Sigma-Aldrich, Inc., St. Louis, MO, USA.	0.1%	X100
		0.2%	
Hydrogen Peroxide (H_2_O_2_)	Sigma-Aldrich, Inc., St. Louis, MO, USA.	0.3%	1085971000
Fetal Bovine Serum	Sigma-Aldrich, Inc., St. Louis, MO, USA.	25%	F7524
		0.1%	
DAB	Sigma-Aldrich, Inc., St. Louis, MO, USA.	manufacturer’s notice	D5905
Trizma^®^ base	Sigma-Aldrich, Inc., St. Louis, MO, USA.	manufacturer’s notice	T1503
Hydrochloric acid (HCl)	Avantor delivered by VWR Radnor, PA, USA	manufacturer’s notice	35328
Masson trichrome with aniline blue staining	Bio-Optica Milano S.p.a Milan, Italy	manufacturer’s notice	04-010802
Sorensen’s Phosphate Buffer, 0.2 M	Avantor delivered by VWR Radnor, PA, USA.	manufacturer’s notice	100496-400
Glutaraldehyde	Sigma-Aldrich, Inc., St. Louis, MO, USA.	2.5%	G5882
Fluoromount Aqueous Mounting Medium	Sigma-Aldrich, Inc., St. Louis, MO, USA.	manufacturer’s notice	F4680

**Table 4:** Antibodies used in this study.

Primary Antibodies	Supplier	Catalog Number	Source	Dilution	Antibody ID
Vimentin RV202	Thermo Fisher Scientific Waltham, MA, USA	OMA1-06001	Mouse	1:100	AB_325529
Calretinin-N18	Santa Cruz Biotechnology Dallas, TX, USA	sc-11644	Goat	1:100	AB_634545
Ubiquitin	Enzo Biochem New York, NY, USA	ADI-SPA-200-D	Rabbit	1:5000	AB_2039666
**Secondary Antibody**	**Supplier**	**Catalogue Number**	**Source**	**Dilution**	**Antibody ID**
Anti-goat IgG (H+L) Alexa Fluor 594	Molecular Probes, Invitrogen, Waltham, MA, USA	A-11058	donkey	1:300	AB_142540
Anti-mouse IgG (H+L) Alexa Fluor 488	Molecular Probes, Invitrogen, Waltham, MA, USA	A-11001	goat	1:300	AB_2534069
Anti-rabbit (IgG) peroxidase-conjugated	Sigma-Aldrich, Inc., St. Louis, MO, USA	A0545	Goat	1:50	AB_257896

## Data Availability

All data presented in this study are available from the corresponding author upon responsible request.

## Supplementary Materials

The following supporting information can be downloaded at: https://www.mdpi.com/article/10.3390/ijms242115619/s1.
