# Supplementary material for: Vimentin Localization in the Zebrafish Oral Cavity: A Potential Role in Taste Buds Regeneration
Source: Int J Mol Sci. 2023 Oct 26;24(21):15619. doi: 10.3390/ijms242115619 (PMC10648301; doi:10.3390/ijms242115619)
Supplement: Supplementary file 1 [file ijms-24-15619-s001.zip › ijms-2665625-supplementary.pdf]

RESULTS

Control experiment of immunoreaction

During the immunofluorescences and immunoperoxidase experiments, the serial representative sections used as a negative control without primary antibodies (Vimentin RV202, Calretinin N-18, and Ubiquitin) showed no immunoreactivity (Figure 11).

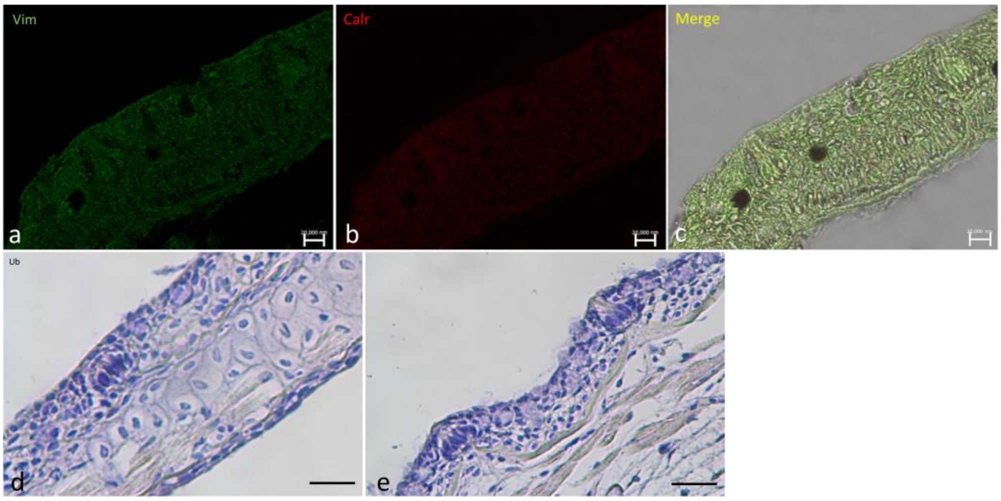

Control experiment performed without primary antibodies showing no immunoreactivity to Vimentin RV202, Calretinin N-18 (**a**, **b**, **c**) and Ubiquitin (**d**, **e**). (**a**, **b**, **c**) Magnification 20×; Scale Bar 20μm. (**d**, **e**) Magnification 20×; Scale Bar 100μm
